# Gene Expression Changes after Parental Exposure to Metals in the Sea Urchin Affect Timing of Genetic Programme of Embryo Development

**DOI:** 10.3390/biology10020103

**Published:** 2021-02-01

**Authors:** Tiziana Masullo, Girolama Biondo, Marilena Di Natale, Marcello Tagliavia, Carmelo Daniele Bennici, Marianna Musco, Maria Antonietta Ragusa, Salvatore Costa, Angela Cuttitta, Aldo Nicosia

**Affiliations:** 1 Institute for Studies on the Mediterranean-National Research Council (ISMED-CNR), Detached Unit of Palermo, Via Filippo Parlatore 65, 90145 Palermo, Italy; tiziana.masullo@cnr.it (T.M.); marilena.dinatale@ismed.cnr.it (M.D.N.); carmelodaniele.bennici@ismed.cnr.it (C.D.B.); marianna.musco@cnr.it (M.M.); 2 Institute for Anthropic Impacts and Sustainability in Marine Environment-National Research Council (IAS-CNR), Detached Unit of Capo Granitola, Via del mare 3, 91021 Campobello di Mazara, Italy; girolama.biondo@ias.cnr.it; 3 Department of Earth and Marine Science (DiSTeM), University of Palermo, Via Archirafi 20, 90123 Palermo, Italy; 4 Institute for Biomedical Research and Innovation-National Research Council-(IRIB-CNR), Via Ugo La Malfa 153, 90146 Palermo, Italy; marcello.tagliavia@cnr.it; 5 Department of Biological, Chemical and Pharmaceutical Sciences and Technologies (STEBICEF), University of Palermo, Viale delle Scienze Ed. 16, 90128 Palermo, Italy; maria.ragusa@unipa.it (M.A.R.); salvatore.costa@unipa.it (S.C.)

**Keywords:** sea urchin, redox homeostasis, parental exposure, intergenerational effects, embryo development, gene expression profiles

## Abstract

**Simple Summary:**

Intergenerational and transgenerational effects, in which exposure to stressors in a parental generation affects the phenotype of the offspring have been connected to anthropic impacts on biological systems. Therefore, environmental stress experienced inside a generation, particularly during gametogenesis, may lead to erroneous patterns in their offspring just emerging at early developmental stages. In this scenario, the sea urchin embryo represents a suitable model for integrating analyses of gene expression through embryogenesis with developmental alteration induced by environmental stressors. Herein we provide pieces of evidence for the alteration of the gene regulatory networks modulating embryo development after parental conditioning via non-lethal metal exposure. We show that the parentals’ conditioning does not affect viability but significantly impairs the developmental fate of the progeny and regulatory network across a generation. It is reasonable to suppose that changes in *Paracentrotus lividus* gonads may modify the expression of regulatory genes modulating synthesis and/or accumulation of maternal determinants, which, in turn, impaired the zygotic activation of GRNs responsible for proper embryo development.

**Abstract:**

It is widely accepted that phenotypic traits can be modulated at the epigenetic level so that some conditions can affect the progeny of exposed individuals. To assess if the exposure of adult animals could result in effects on the offspring, the Mediterranean sea urchin and its well-characterized gene regulatory networks (GRNs) was chosen as a model. Adult animals were exposed to known concentrations of zinc and cadmium (both individually and in combination) for 10 days, and the resulting embryos were followed during the development. The oxidative stress occurring in parental gonads, embryo phenotypes and mortality, and the expression level of a set of selected genes, including members of the skeletogenic and endodermal GRNs, were evaluated. Increased oxidative stress at F_0_, high rates of developmental aberration with impaired gastrulation, in association to deregulation of genes involved in skeletogenesis (*dri, hex, sm50, p16, p19, msp130*), endodermal specification (*foxa, hox11/13b, wnt8)* and epigenetic regulation (*kat2A*, *hdac1,* e*hmt2, phf8* and *UBE2a*) occurred either at 24 or 48 hpf. Results strongly indicate that exposure to environmental pollutants can affect not only directly challenged animals but also their progeny (at least F_1_), influencing optimal timing of genetic programme of embryo development, resulting in an overall impairment of developmental success.

## 1. Introduction

Intergenerational and transgenerational effects, in which exposure to stressors in a parental generation (F_0_) models the phenotype of the offspring (F_1_) [1,2], have been recently reported to be associated with the global climate change and anthropic impacts on biological systems [3,4,5].

Genome- or transcriptome-wide changes induced by environmental stressors on somatic cells can affect the physiology of exposed individuals; however, some alterations can be propagated to subsequent generations through the germline [6]. As such, modifications in the expression of maternal determinants may occur during the gametogenesis, making these alterations heritable [7,8,9]. Therefore, environmental stresses experienced by F_0_, in particular during gametogenesis, may lead to aberrant patterns in F_1,_ which can become evident even since the early developmental stages.

It is known that a network of regulatory genes finely establishes the mechanisms of specification and differentiation through embryo development [10,11]. The eco-devo approach, integrating analyses of gene expression through embryogenesis with developmental alterations induced by environmental stressors, provides a tool for reframing the complexity of embryo development [12,13].

As unique invertebrate deuterostomes, echinoderms and, in particular, sea urchins, represent excellent research models in the study of the developmental biology of bilaterians [14,15], as well as fertilization and proliferation mechanisms [16,17,18] and genome evolution [19]. Moreover, they have been used as models in a lot of ecotoxicological studies for the assessment of the effects of anthropization, ocean warming and acidification [20,21,22,23,24].

The gene regulatory networks (GRNs) that control the sea urchin development represent one of the most complete models integrating embryogenesis and transcriptional regulatory switches [25,26]. Among the studied regulatory circuits, the skeletogenic and the endodermal regulatory networks are well conserved in sea urchin species. In the skeletogenic circuit the transcription factor Ets1, which is maternally inherited, activates the expression of genes encoding the transcription factors Alx1 and Hex. These activate *dri* and the three transcription factors cooperate and activate the expression of genes responsible for calcification and spicule formation, such as *sm50*, *msp130*, *p16* and *p19* [10,27]. Conversely, the endoderm specification starts with the early expression of *wnt8* which turns on the expression of *hox11/13b*, *foxa* and *bra* [10,27].

In addition, developmental programs established by transcription factor networks are promoted, sustained, maintained and transmitted to daughter cells via epigenetic systems, also when the responsible transcription factors are not still active. These systems include post-transcriptional modifications of histone tails by chromatin writers (HAT, HDAC, HKMT, etc.), DNA methylation by DNMTs, long non-coding RNAs, and in turn recruit chromatin readers, such as chromatin remodelers [28,29,30,31]. Any alteration of these cascades may affect chromatin dynamics and deregulate a huge number of genes, causing aberrant developmental phenotypes.

In addition to *Strongylocentrotus purpuratus*, a few species contributed to define the GRNs. Among them, the Mediterranean sea urchin *Paracentrotus lividus* represents a widely studied species [32,33,34]. Interestingly, the high resolved expression profiles of the regulatory genes orchestrating developmental circuits are available also in *P. lividus* and the study of their perturbations due to pollutant exposure represents an important tool also to investigate mechanisms of toxicity [35,36].

The *P. lividus,* as an inhabitant of coastal waters, is exposed to a range of stressors, including pollutants from anthropogenic activities, throughout its life [37].

As a result of its lifestyle, the sea urchin often experiences different environmental conditions which may result in adaptive effects [38,39] and intergenerational effects affecting the phenotype of the offspring [40,41]. In this line, in early-stage *Strongylocentrotus purpuratus* embryos and larvae, high pCO_2_ has been shown to impact body size, metabolic rates, gene expression, internal acid-base balance and growth [42]. Recently, we showed that non-lethal metal conditioning heavily affected the sea urchin transcriptional profiles in reproductive tissues, thus suggesting effects on gametogenesis [43]. Accordingly, gametes from cadmium and manganese-exposed *P. lividus* females experienced difficulties in fertilisation and related embryos displayed abnormalities during the development [40]. Similarly, defects in hatching and larval morphology were also detected in *Strongylocentrotus intermedius* embryos from warm-conditioned F_0_ [44]; however, no data are currently available on the effects that metal exposure of either males or females could exert on the progeny.

Herein, we evaluated if the conditioning of parental *P. lividus*, via metal exposure could affect the developing offspring.

Toward this aim, adult sea urchins were challenged for 10 days with zinc (Zn) as a representative essential metal, and cadmium (Cd), as a representative xenobiotic metal with known toxic effects at environmentally relevant concentrations or similar to those used in other reports [43,45,46]. Since the simultaneous exposure of organisms to different metals may result in variable levels of toxicity due to neutralizing, additive or synergistic effects [22,47], two mixtures, consisting of Cd and Zn at different concentrations, were also used. The metal-conditioned gonads were analysed in order to assess reactive oxygen species (ROS) production and catalase activity, as well as protein levels of HSP70, HSP60 and HSP90.

In order to evaluate the influence of stress events experienced by parentals on the developing progeny, resulting embryos (F_1_) were let to develop until 48 h post-fertilization (hpf), and the morphology was analysed at various developmental stages. As a result that it is known that sea urchin exposure to metals affects the overall developmental regulatory networks, herein we wondered to investigate whether the parental exposure affected selected regulatory nodes. In particular, gene expression was examined in embryos from parents exposed to the low Zn and low metal mixture concentration treatments, because of high mortality rates observed in the other treatments. Therefore, to investigate molecular mechanisms perturbing the development, we analysed the transcriptional profiles of development-related genes, including selected members of the endoderm and skeletogenic GRNs (*foxa*, *hox11/13b*, *wnt8*, *dri*, *hex*, *sm50*, *p16*, *p19*, *msp130*). Moreover, we profiled also the transcriptional expression of genes associated with mechanisms of epigenetic regulation, including histone acetylase and deacetylase (*kat2A* and *hdac1*), histone lysine methylase and demethylase (e*hmt2* and *phf8*) and histone ubiquitinase (*UBE2a*).

Overall, this study begins to unravel the potential for epigenetic mechanisms to play a role in the alterations in the timing or organisation of developmental processes that could also impair the rapid responses to environmental change in an ecologically important benthic marine invertebrate.

## 2. Materials and Methods

### 2.1. Sea Urchin Sampling and Exposure to Metals

Adult *P. lividus* specimens were collected nearby Capo Granitola, Trapani, Italy (Latitude 37°34′30.00″ N Longitude 12°40′47.26″ E) and rapidly transported to the laboratory. All the experimental workflow (including replicates) was performed in autumn/winter 2017. The animals were acclimated for 15 days in an aquarium with artificial seawater (ASW) (Instant Ocean Aquarium System), with 12 h:12 h light: dark photoperiod and continuous aeration. Salinity (35 ± 1‰), temperature (17.0 ± 1.0 °C) and pH (7.80 ± 0.10) were monitored daily. An amount of 3 g/animal of dehydrated macroalgae (Sera Marin Gourmet Nori) was provided every day during the acclimation, while feeding was interrupted 2 days before experimental sampling. After acclimation, *P. lividus* adults (3 males and 3 females per treatment) were exposed for 10 days to cadmium, zinc and combinations of the two pollutants in different tanks (10 L each) as reported in Table 1. Specimens in ASW were used as controls. Temperature and pH values were monitored daily. Each experiment was carried out three times. Each animal was treated just once, therefore the total number of males and females investigated in the experiments was 126 (3 males × 7 conditions (6 treatments + 1 control) × 3 replicates = 63 males treated plus 3 females × 7 conditions × 3 replicates = 63 females treated). Sulphate salts (Sigma-Aldrich, Milan, Italy) were used for the preparation of metal solutions. During exposure, animals were fed twice a week with 3 g/animal of dehydrated macroalgae.

### 2.2. Protein Extraction, SDS-PAGE and Western Blot Analyses

Male gonads were pooled (0.3 g) and homogenized in 20 mM Tris–HCl buffer pH 7.5, 15% Glycerol, 1 mM EDTA, 0.1% Triton X-100. Similarly, 0.3 g of female gonads were also pooled and treated in the same manner. Therefore, a total of 42 samples (3 replicates/condition for males; 3 replicates/condition for females; 7 conditions) were analysed. The homogenates were centrifuged to remove any insoluble debris at 10,000 rpm for 10 min. The supernatants were collected and quantified using Qubit 2.0 Fluorometer (Thermo Fisher, San Jose, CA, USA) according to the manufacturer’s instructions and protein concentration was brought to 2.5 mg/mL in all samples. Proteins (50 µg) were separated on 10% SDS-PAGE under reducing conditions and transferred to nitrocellulose membranes (Thermo Fisher, CA, USA) at 50 V, in ice bath, for 2 h using transfer buffer (48 mM Tris, 39 mM glycine, pH 9.2, 20% methanol). After blocking in 5% BSA and 1× TBST (20 mM Tris-HCl pH 7.6, 137 mM NaCl, 0.1% Tween 20) for 1 h at room temperature, membranes were incubated overnight at 4 °C with different antibodies. In particular, monoclonal anti-Heat Shock Protein 70 (H5147, Sigma, Milan, Italy), monoclonal anti-Heat Shock Protein 60 (H-3524, Sigma, Milan, Italy), monoclonal anti-Heat Shock Protein 90 (SAB1305541, Sigma, Milan, Italy) and monoclonal anti-actin clone (A3853, Sigma, Milan, Italy) primary antibodies, were used and diluted 1:2000, 1:500, 1:800 and 1:500, respectively, in 1× TBST. The membranes were washed three times with TBST for 5 min and incubated for 1 h at room temperature with alkaline phosphatase-conjugated anti-mouse IgG (1:3000). The membranes were washed three times for 5 min with TBST and incubated with One step NBT/BCIP substrate solution (Thermo Fisher, CA, USA) and blots were acquired using the Molecular Imager VersaDoc system (Bio-Rad Laboratories, Hercules, CA, USA). The Image J software (Bio-Rad Laboratories) was used for densitometry analysis of the immunoblotted bands. The signals from each protein band were normalized against the β-actin content.

### 2.3. Reactive Oxygen Species (ROS) Detection and Catalase Activity

The catalase assay kit (CAT 100, Sigma, Milan, Italy) was used to measure the catalase activity in protein extracts. A standard curve was generated plotting the Red Quinoneimine dye absorbance at 520 nm versus different amounts of H_2_O_2_ (0.0125, 0.025, 0.05 and 0.075 µ/mol). Supernatants were added to the catalase colorimetric enzymatic reaction to determine the amount of H_2_O_2_ remaining in the mixture using the H_2_O_2_ standard curve. In the assay reaction, a volume amount corresponding to 20 µg of total protein content for each sample was added.

In particular, Equation (1) was used to calculate the difference in the amount of H_2_O_2_ (∆µ/moles) added to the colorimetric reaction between the blank and a given sample, while the catalase activity was calculated using the Equation (2).

∆μ/moles (H_2_O_2_) = µ/moles of H_2_O_2_ (blank) − µ/moles of H_2_O_2_ (sample) (1)

Activity (µ(moles/min)/mL) = ∆μ/moles (H_2_O_2_) × d × 100)/(V × t) (2)

where d is the dilution of the original sample for catalase reaction; t is the catalase reaction duration (min); V is the sample volume in catalase reaction (mL); 100 is the dilution of an aliquot from catalase reaction in colorimetric reaction. To measure total ROS/RNS free radical activity present in *P. lividus* reproductive tissues the OxiSelect In Vitro ROS/RNS Assay Kit (Cell Biolabs, Inc. San Diego, CA, USA) was used according to the manufacturer’s instructions. Supernatants were incubated in a 96-well fluorescence plate with the DCFH probe to allow the oxidation reaction to proceed. Samples were then measured fluorometrically at λex 480 nm and λem 530 nm using the GloMax Discover Multimode Microplate Reader (Promega, Milan, Italy). The free radical content in samples was determined by comparison with a DCF standard curve (1 nM–10 µM).

### 2.4. Embryo Culture and Morphological Analysis

Exposed and control *P. lividus* animals were sacrificed and gonads were gently washed to allow gametes collection. In particular, for each experimental condition, eggs from treated females (3 females/treatment/experiment) were pooled and maintained in ASW until fertilisation with sperms (3 males/treatment/experiment) collected and diluted (1:500) in ASW. Similarly, pooled eggs from three untreated animals were fertilised in presence of pooled sperms from control males. All the performed fertilisation occurred in the condition of sperms/eggs ratio corresponding to 100:1. Eggs were rinsed after fertilization. At least 6000 embryos were allowed to develop in 250 mL tanks at 18 °C under gentle agitation in ASW and they were examined until the pluteus stage (48 hpf). The percentages of embryos showing normal or abnormal phenotype were determined by counting about 100 embryos/replicate in each treatment using the Olympus BX50 optical microscope.

### 2.5. RNA Purification and First-Strand cDNA Synthesis

Total RNA was purified from *P. lividus* embryos from parentals treated with the low concentration of Zn and the low concentration of the metal mixture (1000 embryos/treatment/experiment), at 24 and 48 hpf, using TRIzol Reagent (Invitrogen Corporation, Carlsbad, CA, USA) according to the manufacturer’s instructions. RNA concentrations and purity (Abs260, Abs260/280 and Abs260/230) were spectrophotometrically verified using Eppendorf 6131 BioPhotometer (Eppendorf AG, Hamburg, Germany). RNA integrity was evaluated on 1.5% agarose denaturing gel and RNA was stored at −80 °C for future use. An amount of 1 µg of total RNA was digested with Deoxyribonuclease I, Amplification Grade (Invitrogen Corporation, Carlsbad, CA, USA) to remove genomic DNA contamination, while DNase I was inactivated by adding 25 mM EDTA. First-strand cDNA was synthesised from 500 ng DNase I treated RNA using SuperScript VILO cDNA Synthesis Kit (Invitrogen Corporation, Carlsbad, CA, USA), according to the manufacturer’s instructions. The synthesised cDNAs were tested by PCR using *18S* rRNA (Table 2) and then diluted 1:10 before use in real-time qPCR experiments.

### 2.6. Gene Expression Profiling by Real-Time Quantitative Polymerase Chain Reaction (qPCR)

The qPCRs were carried out on the BIO-RAD CFX96 system using Power Sybr Green as the chemical detection method (Applied Biosystems, Forster City, CA, USA). The qPCRs were performed in 96-well plates in a 20 µL mixture containing 1 µL of a 1:10 dilution of the cDNAs, using the following PCR parameters: 95 °C for 10 min, followed by 40 cycles of 95 °C for 10 s and 60 °C for 35 s and melting curve from 65 to 95 °C. Amplicons were run on an agarose gel to confirm the specific gene amplification.

The *18S* ribosomal RNA was chosen as reference gene [43,48,49,50,51]. PCR efficiency of the target and reference genes was calculated using serial dilutions of pooled cDNAs from both control and treated samples. The measured amplification efficiency ranged from 1.8 to 2.1. Primer sequences used in this study are listed in Table 2.

Quantitative real-time PCRs were conducted according to the manufacturer’s recommended procedures, and every reaction was performed three times. Data analysis was carried out according to the 2^−ΔΔCT^ method [52].

### 2.7. Statistical Analysis

**Figure 1:**
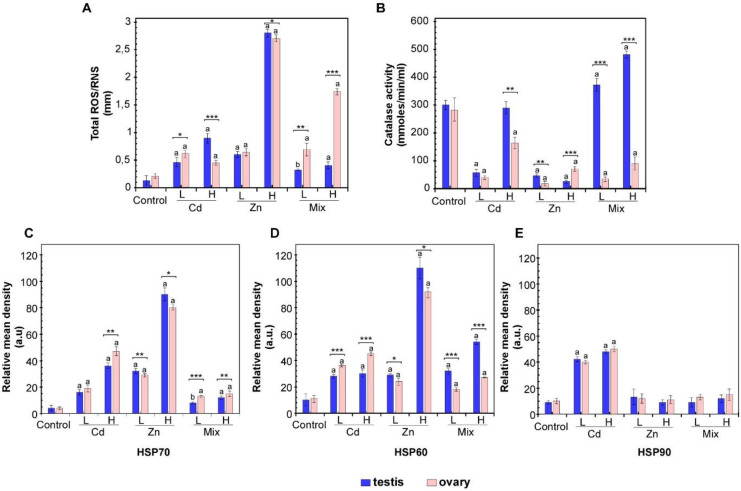
Redox response system of *P. lividus* reproductive tissues (testis and ovary) after exposures to high (H), low (L) doses of Cd, Zn or a mix of both metals, as reported in Table 1. (**A**) Total ROS amount; (**B**) catalase activity; (**C**) relative HSP70 protein amount; (**D**) relative HSP60 protein amount; (**E**) relative HSP90 protein amount. Bars represent the mean values from the three different experiments ± SD. Statistical significance among testis and ovary results is shown as asterisks (*: *p* < 0.05; **: *p* < 0.01; ***: *p* < 0.001). Letters denote statistical significance of treated specimens vs. controls (b: *p* < 0.01; a: *p* < 0.001).

**Figure 2:**
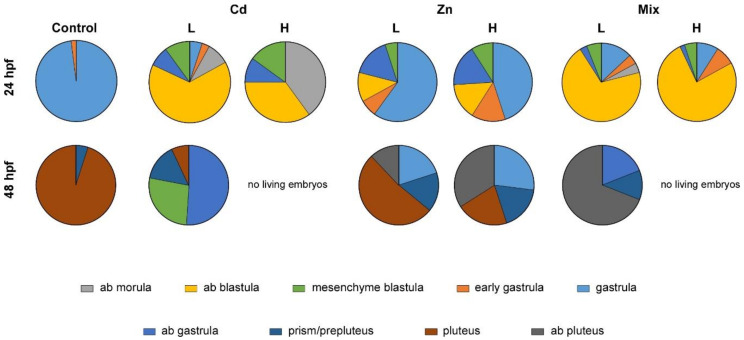
Embryo morphological abnormalities at 24 and 48 hpf and percentages in response to the different treatments (Cd, Zn and Mix of them) experienced by parentals. Data corresponding to embryos from means of three experiments are shown; SD were lower than 5%. L: low concentration; H: high concentration; Ab: abnormal.

Experiments were performed in triplicate. The results in the bar plots were expressed as a mean value ± SD. To compare treatment results in testis and ovary, ANOVA tests were performed using the R software, version 3.6.3 (Figure 1). Statistical analysis on phenotype evaluation (Figure 2) was done using *t*-test. For gene expression analysis, significant differences between the values of different treated groups and the reference control groups were also determined by *t*-test. The *p*-values less than 0.05 were considered statistically significant.

## 3. Results

### 3.1. Effects of Metal Exposures on the Stress Response of P. lividus Reproductive Tissues

*P. lividus* specimens were exposed to Cd, Zn and a mixture of both metals, each in two different concentrations, low and high for 10 days as described in the Material and Methods. In F_0_, no mortality or illness indicators, as dropped spines or reduced movements, were recorded among sea urchins during metal conditioning.

In order to evaluate the stress status of challenged *P. lividus* gonads, the production of ROS was evaluated in exposed and control animals. As shown in Figure 1A, a significant increase in ROS accumulation in male and female gonads was induced by metal exposure. Zn treatments resulted in a concentration-dependent increase in ROS contents in the testis and ovary. Evidence for gender-specific responses emerged for ROS production induced by metal mixes because ROS level increased more in ovaries than in testis.

The activity of catalases (CAT) was also profiled in order to evaluate the activation of the antioxidant system involved in ROS scavenging. As expected, catalase activity was inversely correlated with ROS levels. An overall reduction in CAT activity was found in female reproductive tissues in response to different Cd and Zn doses, as well as after combined exposures. A similar pattern was also observed in the testis, except for the unchanged CAT activity measured at the higher Cd dosage. Additionally, increased CAT activity in testis was measured in response to Mix L and Mix H (Figure 1B).

To evaluate the effects of metal-induced stress at the protein scale, we analysed the expression of HSP70, HSP60 and HSP90 proteins (Figure 1C–E and Supplementary Materials Figure S1). The densitometric analysis showed a concentration-dependent overexpression of HSP70 protein in male and female gonads exposed either to Cd and Zn (Figure 1C); while, evidence for gender-specific responses emerged for HSP60. In detail, although metal-induced HSP60 synthesis was found both in testis and ovarian tissue, exposure to increasing Cd and Zn levels resulted in dose-dependent HSP60 overexpression in the ovary; differently, in testis HSP60 upregulation was dose-dependent only in response to Zn (Figure 1D). For HSP90 increased levels were found in response to Cd, while Zn treatment did not induce significant changes (Figure 1E). To evaluate the effects of the combined exposures, the expression of these stress-related proteins was also measured in sea urchins exposed to Mix H and Mix L (Figure 1C–E). Densitometric analysis revealed that co-exposures induced HSP60 and HSP70, although to a lesser extent than observed with single metals. Moreover, albeit HSP60 increased especially in testis, HSP70 induction were higher in ovary. Conversely, HSP90 resulted uninduced.

### 3.2. Effects of Metal Exposures on the Offspring of Conditioned P. lividus

To evaluate the occurrence of intergenerational effects due to metal exposures of parentals (F_0_), eggs and sperms from exposed animals were allowed to fertilise and develop until 48 hpf (which corresponds to the pluteus stage in controls). No significant differences in fertilization success were observed across experiment conditions, and embryos were observed under a microscope at 24 and 48 hpf, corresponding to the gastrula and pluteus stages, respectively (Figure 2 and Supplementary Materials Figure S2).

Embryos from Cd (L) conditioned parentals showed several abnormalities. Retard in endoderm invagination occurred at 24 hpf and a high percentage of embryos (75%) were unable to proceed beyond the blastula stage. At 48 hpf abnormal blastulae, gastrulae and prepluteus stage embryos occurred (88%).

The high Cd concentration dramatically affected the development of the progeny. At 24 hpf, embryos showed abnormal development with blastomeres defects. Additionally, several embryos did not gastrulate and showed altered archenteron invagination. Moreover, apparently, there were no living embryos in the culture. Indeed, at 48 hpf, embryos completely degenerated.

The exposure of parentals to Zn also caused abnormality in the offspring. However, the percentages of abnormal embryos observed were lower than embryos from Cd conditioned gametes. At 24 hpf the 33% of embryos from Zn (L) conditioned parentals showed problems in gastrulation, such as abnormal endoderm invagination. Moreover, at 48 hpf (pluteus stage in the controls) developmental defects and skeletal malformations were more evident and variable morphologies were obtained. A significant number of embryos showed defects in spicule formation or elongation resembling gastrulae, prisms or plutei with arms shorter than controls. However, the mortality rate remained lower than Cd (L) exposed parentals. As expected, abnormalities were more severe in the offspring of parentals who experienced high Zn dosage either at 24 and 48 hpf.

Combined exposure to Cd and Zn (Mix L) caused significantly higher percentages of abnormalities (83% abnormal blastulae at 24 hpf) with respect to the treatment with Cd or Zn only. Living embryos displayed the worst phenotype, resulting unable to gastrulate and showing abnormal segmentation. Blastomeres were poorly connected and several isolated cells appeared in the background. At 48 hpf, significant increase in degenerated embryos occurred; additionally, shortened plutei with fractured ectoderm and incomplete skeletal rods were also present. Apparently, despite the aberrant phenotypes, the mortality rate was relatively low; conversely, no living embryos could be detected in the offspring of parentals who experienced Mix H.

Overall, parental exposure to metals can have profound consequences on gastrulation timing and embryo morphology.

### 3.3. Pathway-Centered Gene Expression Profiling

**Figure 3:**
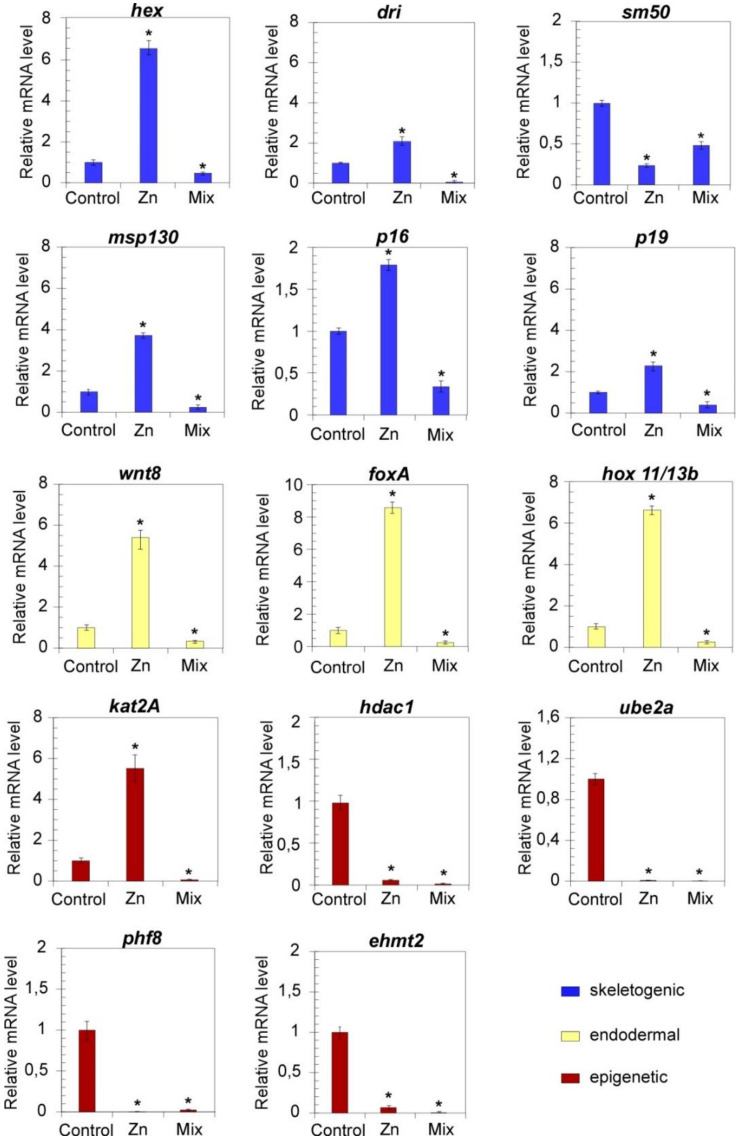
Parental exposures to low doses of Zn and metal mixture (as defined in Table 1) induce alterations in the mRNA expression of development and epigenetic related genes at 24 hpf. RT-qPCR results showing the mRNA levels of indicated genes in *P. lividus* offspring, with respect to *18S* at 24 hpf. Bars represent mean ± SD. Values were considered statistically significant at *p* less than 0.05 (*).

**Figure 4:**
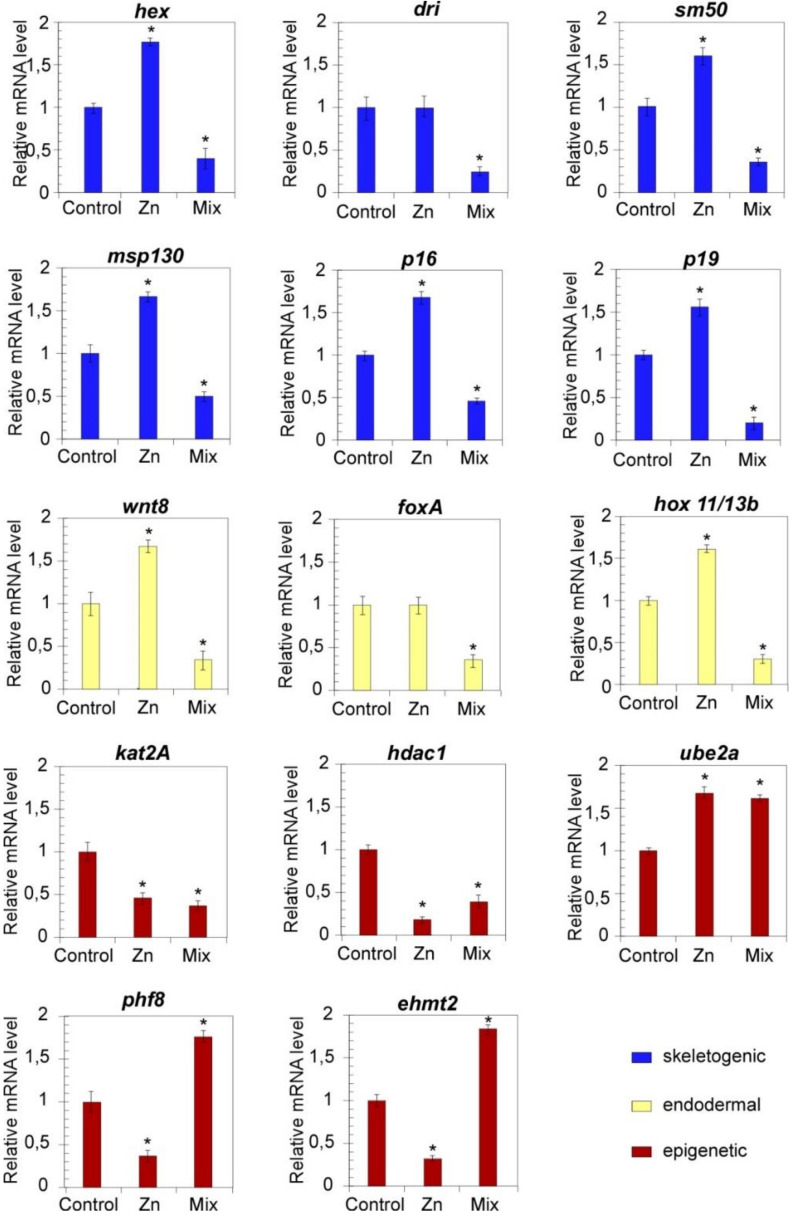
Parental exposures to low doses of Zn and metal mixture (as defined in Table 1) induce alterations in the mRNA expression of development and epigenetic related genes at 48 hpf. RT-qPCR results showing the mRNA levels of indicated genes in *P. lividus* offspring, with respect to *18S* at 48 hpf. Bars represent mean ± SD. Values were considered statistically significant at *p* less than 0.05 (*).

In the light of the recognized roles of precise genes in mechanisms of gene regulation and chromatin remodelling, we performed a pathway-centred gene expression analysis on developmental regulators of skeletogenic (dri, hex, sm50, p16, p19, msp130) and endodermal differentiation (foxa, hox11/13b, wnt8) [10,27], as well as on epigenetic regulators of chromatin accessibility (kat2A, hdac1, ehmt2, phf8 and UBE2a) (Figure 3 and Figure 4). The high deformity rate (higher than 50%) observed among the offspring of parentals who experienced Cd (L and H), the highest dosage of Zn and their combinations prompted us to analyse the transcriptional effects exclusively at the lowest Zn and Mix metal concentration whose low mortality rate do not expect to affect the RT-qPCR results.

#### 3.3.1. Expression Profiles of Development Regulators

In order to unveil possible variations in the transcriptional profile of members of the *P. lividus* GRNs, the mRNA levels of *hex*, *dri*, *sm50*, *msp130*, *p16*, *p19*, *wnt8*, *foxa* and *hox11/13b* were analysed in embryos (F_1_) from exposed parentals. The results are shown in Figure 3 (24 hpf) and Figure 4 (48 hpf).

The expression of all the genes was largely affected in embryos from Zn-conditioned parentals and a huge reduction of mRNA levels was measured in response to specific exposures. Except for the downregulation of *sm50*, the F_1_ of Zn-conditioned animals showed an overall transcriptional upregulation. Indeed, the RT-qPCR analysis revealed that the mRNA expression levels of the selected genes increased significantly in such embryos at 24 hpf with respect to untreated embryos. Even at 48 hpf, the general expression levels were still higher than controls albeit to a lesser extent, with a few exceptions, including dri and foxA, whose mRNA levels returned similar to controls. The mRNA levels of these genes were also evaluated in the offspring of animals exposed to Mix L. The qPCR analyses revealed that coexposure negatively affected the expression of examined transcripts since all the mRNA resulted downregulated both at 24 and 48 hpf.

#### 3.3.2. Expression Profiles of Transcriptional Regulators

To evaluate the perturbation affecting the mRNA levels of genes involved in mechanisms of chromatin remodelling, the transcriptional profile of *kat2A*, *hdac1*, *UBE2a*, *phf8* and *ehmt2* was also analysed (Figure 3 and Figure 4).

At 24 hpf (Figure 3), the F_1_ from Zn conditioned sea urchins showed a significant general reduction in the transcriptional levels of the studied genes. However, upregulation events were also measured, since *kat2A* increased at 24 hpf (Figure 3), while significantly increased *UBE2a* mRNA levels were measured at 48 hpf (Figure 4).

To probe the effects of joint metal exposures, the transcriptional levels of this gene panel was also profiled in embryos from adults exposed to Mix L. Once again, a general transcriptional downregulation was observed at 24 hpf. Differently, *ehmt2*, *phf8* and *UBE2a* resulted upregulated at 48 hpf.

## 4. Discussion

It has been reported that sea urchins can accumulate metals and they have been used as indicators of environmental pollution [53]. However, data reporting correlation between the environmental level of toxicants and accumulated metals do not comply with this intent. In particular, Cd and/or Zn content in *P. lividus* gonadal tissue from contaminated areas often does not reflect the environmental levels, as it does not result higher than that found in animals from low anthropic impact areas [54,55,56]. However, the possibility of effects on the offspring has been observed. In order to provide pieces of evidence on the transmission of parental stress to the progeny at the phenotypical and transcriptional level, we evaluated the effects of prolonged and non-lethal metal exposures on *P. lividus* adult individuals and the related F_1_. Recently, we have shown that sub-lethal metal exposures dramatically affect the canonical transcriptional profiles in reproductive tissue which, in turn, could alter spermatogenesis and oogenesis [43]. Herein we show that the exposure to Cd, Zn and their mixes induces an increase in ROS production similarly to those reported for different systems [57,58,59,60]; accordingly, catalase activity showed variations, presumably related to its role in the maintenance of the redox homeostasis, especially after Cd exposure. However, despite the increase in ROS levels, CAT activity was reduced in male and female gonads in response to Zn. These results are unusual [61,62,63] and suggest that other enzymes could be involved in the maintenance of the redox homeostasis. In response to mixes, CAT activity appears to reflect the amount of generated ROS so as to cope with the generated level of toxicity. In accordance with previous data on the specific gene expression pattern observed in testis and ovary [43], evidence for gender-specific responses to ROS emerged. Differences in stress susceptibility and tolerance to stress among sexes have been described elsewhere [64]; thus, it could be hypothesised that mechanisms of gonad maturation and gametogenesis may be likely responsible for the different responses.

Beyond this, a reduction in antioxidant activities was also reported in various organisms after metal exposure [65,66]. In this line, it appears that the redox response system of the sea urchin reproductive tissues may result impaired above certain ROS levels in response to experienced levels of toxicity. However, it is also conceivable that the prolonged treatments may result in possible acclimation and tolerance to stressors. In order to assess such an issue, we made efforts in the evaluation of the protein level of HSP60, HSP70 and HSP90. The HSPs exert a protective role as chaperones, by assisting protein folding and preventing their aggregation also in response to stressors including ROS, warming and metals [67,68,69,70,71]. Accordingly, their upregulation confirms the activation of a defence mechanism in the *P. lividus* gonads, which act to cope with the stress so as to tolerate, at least, the rate of ROS. Moreover, the pattern observed in response to mixes suggests that the coexistence of diverse toxicants simultaneously activating different signalling pathways may result in HSPs profiles different from those arising in response to a single contaminant.

It has been shown that the environmental stress experienced by parentals during gametogenesis may result in phenotypic changes of the offspring [72,73]. In this line, it has been reported that sea urchin maternal exposure to different conditions, including acidification and polycyclic aromatic hydrocarbons, affects the capacity of embryos to counteract oxidative stress conditions [74,75,76].

These findings prompted us to evaluate the possible occurrence of embryo defects originating from Cd and Zn conditioned parentals (both male and female sea urchin adults). Although Cd and Zn parental exposure diversely affected gonads, herein we show that the related offspring displayed significant difficulties in proceeding through the canonical developmental program. Additionally, combined metals exposure dramatically affected the fitness of the progeny, given the higher percentages of abnormalities and developmental failure rates recorded using the higher dosage. It appears that a common effect was a delay in cell differentiation and gastrulation events, probably caused by alterations of timing of specific pathways resulting in the abnormal relative order of events that could disrupt spatial relationships or signalling interactions and cause different aberrant phenotypes.

As a result of such phenotypic alterations, it was intriguing to profile the gene expression pattern of key factors that are responsible for the molecular mechanisms orchestrating the regulatory network of embryo development. It is well-known that accurate control of gene expression is mandatory for normal development. As a general rule, in the *P. lividus* skeletogenic lineage, the maternally inherited transcription factor Ets1 activates the expression of several genes including *alx1*, *hex* and *dri* which, in turn, cooperate to activate the expression of terminal differentiation genes, including the spicule matric gene *sm50*, *msp130*, *p16* and *p19* [10,27]. It has been reported that several stressors, including metals [77], X-rays [78], decadienal [79] and UVB [80] affect the expression of the skeletogenic genes.

Interestingly, we show that similar changes occurred also in the F_1_ of exposed animals (Figure 3 and Figure 4). In this line, it is reasonable to suppose that upregulation of *dri* and *hex,* as measured in the embryos from Zn exposed parentals, may rely on modifications in the pattern of maternal determinants that normally occur during gametogenesis. According to *hex* and *dri* aberrant expression, *msp130*, *p16* and *p19* resulted upregulated probably due to an excess of stimuli for gene activation, which may likely result in the observed skeleton formation in embryos. Beyond the amplitude of mRNA levels, it should be noted also the deregulation of *sm50* expression timing. Typically, the architecture of a gene regulatory circuit activating *sm50* is defined as a coherent feedforward structure based on Hex and Dri [27]. Although the upregulation of *dri* and *hex* occurred at 24 hpf a delay in the expression of *sm50* was measured. Thus, it is reasonable to hypothesise that the exposure to metals at F_0_ affects the genetic program of F_1_ in terms of measured mRNA level and timing of developmental programs.

Similarly, the fine-tuning of endodermal signalling, based on the activation of the *wnt8-hox11-13b-foxa* node [81], were downregulated, which also explains the observed defects in the endoderm invagination.

It has been reported that several genes of the sea urchin embryo regulatory network are affected by Zn treatment both spatially and quantitatively. Among them, *hex*, *p19* and *wnt 8* were upregulated in *S. purpuratus* treated embryos, thus providing evidence that Zn perturbs the pattern of endomesoderm and skeletogenic signalling [82]. Therefore, it is reasonable to assume that the defects observed in the progeny of Zn-exposed parentals could be explained by the same mechanisms which could exert adverse effects on the regulatory networks of differentiation.

Interestingly, Zn and Cd co-exposure of parentals abrogated the transcriptional induction of all the analysed genes. These data confirm once again that the co-occurrence of different stressors, triggering multiple pathways, may provide gene expression profiles that were not overlapping to those occurring in response to a single contaminant also via an inherited manner.

Recently, several lines of evidence suggested that maternal exposure to different conditions affected the DNA methylation status of the offspring [83,84]. In particular, several genes involved in mechanisms of transcriptional regulation, including DNA modification and metabolism, as well in protein ubiquitination, were identified as differentially methylated in the offspring. Epigenetic regulation through histone modification may be altered by several environmental factors, including metals, that can affect histone and DNA writers and therefore influence the pattern of acetylation/methylation/ubiquitination of histones and DNA methylation [85,86,87,88]. Although these systems were not studied thoroughly in sea urchins, other models, including mouse embryonic stem cells, provide evidence for H3K27me1 reduction following exposure to different metals, including cadmium [89]. Therefore, it is not surprising that the genes involved in chromatin remodelling, as well as epigenetic regulators (*kat2A*, *hdac1*, *ehmt2*, *phf8* and *UBE2a*), showed huge modifications in the expression profiles in the progeny from conditioned parentals, irrespective of the toxicity levels or experienced metals.

Additionally, it should be noted that, despite the transcriptional activation of GRN developmental regulators, yet at 24 hpf, the observed embryo phenotypes do not correlate with the molecular profiles. Thus, it appears that triggered specification and developmental processes are unable to be timely maintained and finalised probably due to the altered expression profiles of epigenetic regulators.

## 5. Conclusions

In the last years, a great deal of interest has been generated in the characterization of the phenotypic alterations in the offspring from parentals that had experienced environmental stresses, especially during gametogenesis. Herein, we show that F_0_ conditioning that did not affect the viability of the exposed individuals, significantly impaired the developmental fate of the progeny and altered various regulatory gene networks across a generation. It is reasonable to suppose that changes in *P. lividus* gonads may modify the expression of specific gene sets required for synthesis and accumulation of maternal determinants which, in turn, impaired the zygotic activation of GRNs responsible for proper embryo development. Further research should address whether maternal or paternal exposures could differentially affect germ cells and thus embryo development. Similarly, comparison of epigenetic landscape among generations and studies on the localisation of maternal determinants should provide novel insights in the fine mechanisms integrating embryo physiology and environment.

## Figures and Tables

**Table 1:** Concentrations of considered metals.

Treatment	Low (µg/L) L	High (µg/L) H
Cd^2+^	10	100
Zn^2+^	40	200
Mix	Cd-Zn (10 + 40)	Cd-Zn (100 + 200)

**Table 2:** Genes and oligonucleotide primers used in this study.

Gene Name	Gene Symbol	Primer Sequence (5′–3′)
Forkhead box transcription factor a	*foxa*	CAGGTATGGGAAGCATGGGA GCGTATCTCATCGACATGGC
Homeodomain containing transcription factor Hox11/13b	*hox11/13b*	TTGCGACGTTCACAACAACT GTGAGATCGAGAGCCTGTGA
Wint8	*wnt8*	AAGTGTCATGGCGTCTCTGG ATGAGCTTGCCACTGACGAA
Deadringer-like	*dri*	TGGAAATAGACGAGAGGGGC GTGGTATCATGGTGGGTGGA
Hex homeodomain transcription factor	*hex*	TGAACCACCCTACTCCACTG GCCGCTCTATTTTGTCCGAG
Spicule matrix protein 50	*sm50*	GATGGCACACCAGCTTATCC CTGACGCTTCATGACTGGAG
Biomineralization protein p16	*p16*	AGCAGGAGCAGTCGGAGATAC CATCATCACTTCCCATATCGC
Biomineralization protein p19	*p19*	AGAGACCAGGCAGGAGACTAAG GTTGATGTCGAGCTTGTCTTTC
Mesenchyme-specific cell surface glycoprotein	*msp130*	ATACATGGCAACCCAAGAAG CGATTCCAACGAAGATGAGT
Ubiquitin-conjugating enzyme E2 A	*UBE2a*	GATTTGAGGAGTGGAGGATTG AGCTGGCGGATCTTCTTGTA
Histone deacetylase	*Hdac1*	TCACGCCAAGAAGTCAGAAG CCGTGGTGGATATCAATGTC
Histone acetyltransferase	*kat2A*	TGCAATGGATGGAAGAACC ATGTGCAGCAAGTTGATGG
Histone-lysine N-methyltransferase	*EHMT2*	GTGCAGGAGCTCTTGGTAATG ACAGAGAGGGTCGGAAAGTG
Histone lysine demethylase	*phf8*	AGCAGTTGCCATTCCTTTTC CGACCCATTCACATTCCAC
18S ribosomal RNA	*18s*	GAATGTCTGCCCTATCAACTTTCG TTGGATGTGGTAGCCGTTTCTC

## Data Availability

The data herein presented are available in this article and Supplementary Material.

## Supplementary Materials

The following are available online at https://www.mdpi.com/2079-7737/10/2/103/s1, Figure S1: original Western blot results for HSP70, HSP60, HSP90 expression in testis and ovary. Figure S2: phenotypes of *P. lividus* embryos from conditioned parentals.
