# Supplementary material for: Gene Expression Changes after Parental Exposure to Metals in the Sea Urchin Affect Timing of Genetic Programme of Embryo Development
Source: Biology (Basel). 2021 Feb 1;10(2):103. doi: 10.3390/biology10020103 (PMC7912929; doi:10.3390/biology10020103)

Developmental and gene expression changes after parental exposure to metal in the sea urchin

Tiziana Masullo[1§] Girolama Biondo[2§], Marilena Di Natale[1,3§], Marcello Tagliavia[4], Carmelo Daniele Bennici[1], Marianna Musco[1], Maria Antonietta Ragusa[5], Salvatore Costa[5], Angela Cuttitta[1*] and Aldo Nicosia[4*]

[1]National Research Council-Institute for studies on the Mediterranean (ISMED-CNR), Detached Unit of Palermo, Via Filippo Parlatore, 65, 90145 Palermo; tiziana.masullo@cnr.it, marilena.dinatale@ismed.cnr.it, carmelodaniele.bennici@ismed.cnr.it, marianna.musco@cnr.it, angela.cuttitta@cnr.it

[2]National Research Council-Institute for Anthropic Impacts and Sustainability in Marine Environment (IAS-CNR), Detached Unit of Capo Granitola, Via del mare 3, 91021 Campobello di Mazara (TP), Sicily, Italy; girolama.biondo@ias.cnr.it

[3]Department of Earth and Marine Science (DiSTeM), University of Palermo, Via Archirafi 20, 90123 Palermo, Italy; marilenavita.dinatale@unipa.it

[4]National Research Council-Institute for Biomedical Research and Innovation (IRIB-CNR), Via Ugo La Malfa 153, 90146 Palermo, Italy; aldo.nicosia@cnr.it, marcello.tagliavia@cnr.it;

[5]Department of Biological, Chemical and Pharmaceutical Sciences and Technologies (STEBICEF), University of Palermo, Viale delle Scienze, Ed. 16, 90128, Palermo, Sicily, Italy; salvatore.costa@unipa.it, maria.ragusa@unipa.it

*Correspondence: aldo.nicosia@cnr.it (A.N.); angela.cuttitta@cnr.it (A.C.)
[§]These authors contributed equally to this work

Supplementary file
(Fig.S1 and S2)

Figure S1. original Western blot results for HSP70, HSP60, and HSP90 expression in testis and ovary referred to Figure 1 C, D, E.

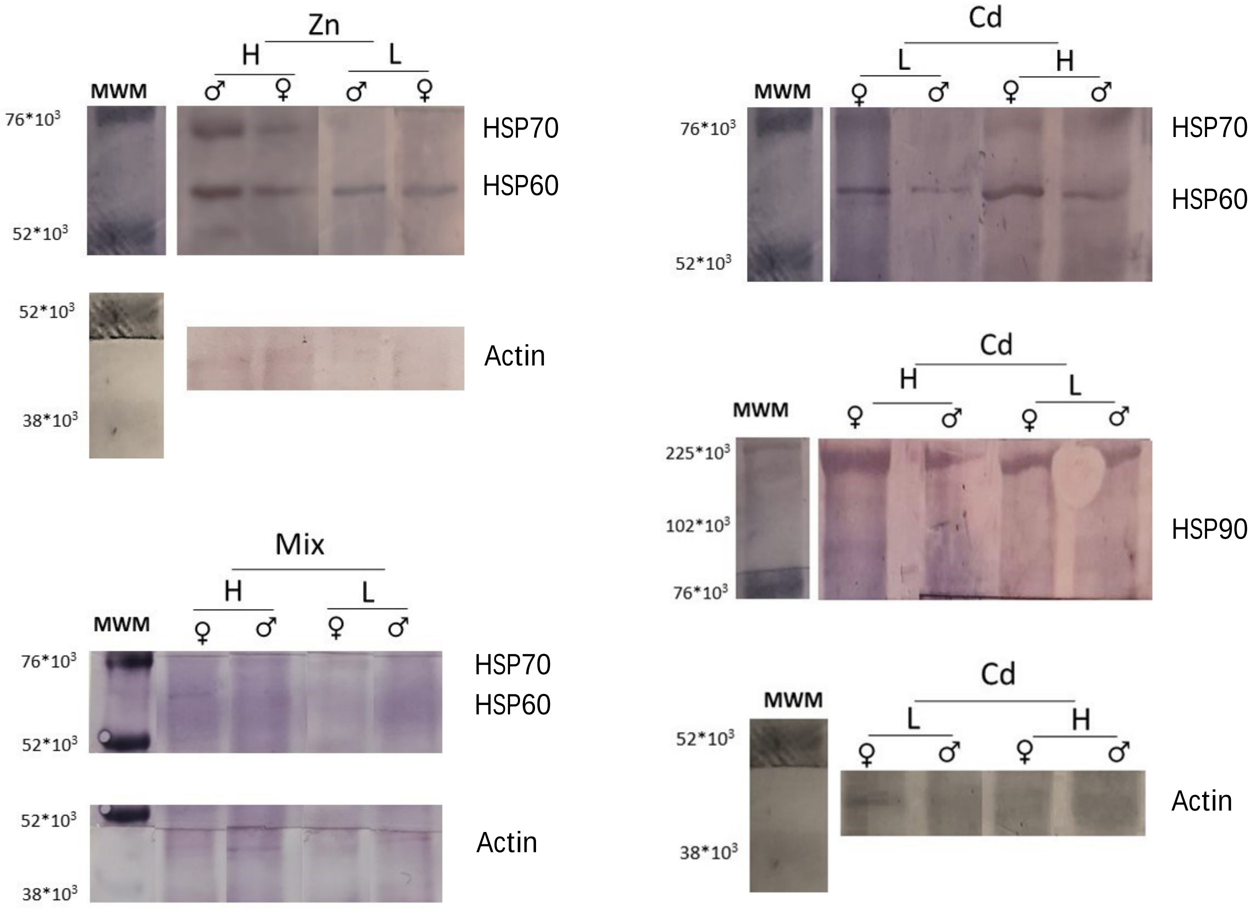

Figure S2. *P. lividus* embryos from parentals exposed to Cd, Zn and Mix of the two metals at 24 and 48 hpf.

Typically, 24 hpf gastrulae show archenteron (a short stub-like gut rudiment) formed and extended until at least halfway. In early gastrulae a ring-like arrangement of PMCs (primary mesenchyme cells) is formed, surrounding the invaginating archenteron but the archenteron invagination is still starting. Normally, the PMCs are not uniformly distributed but assemble to form spiculogenic syncytia. Abnormal gastrulae (more or less early) show altered archenteron invagination and/or altered patterns of PMCs distribution in the blastocoel. Later in development, this causes abnormal skeletons and altered shapes of larvae, that we categorized as "abnormal pluteus".

P.lividus embryos from parentals exposed to Cd L at 24hpf

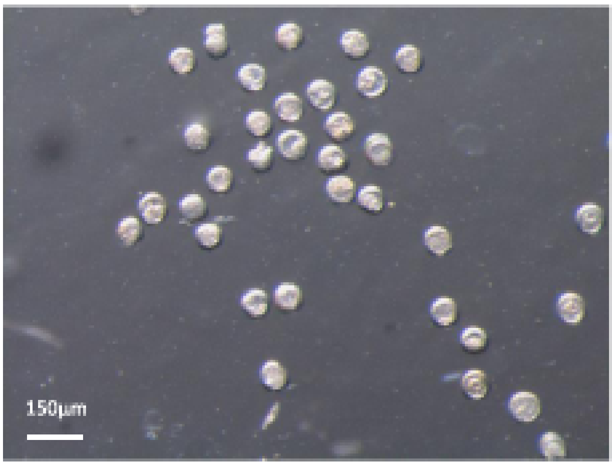

P.lividus embryos from parentals exposed to Cd L at 48 hpf

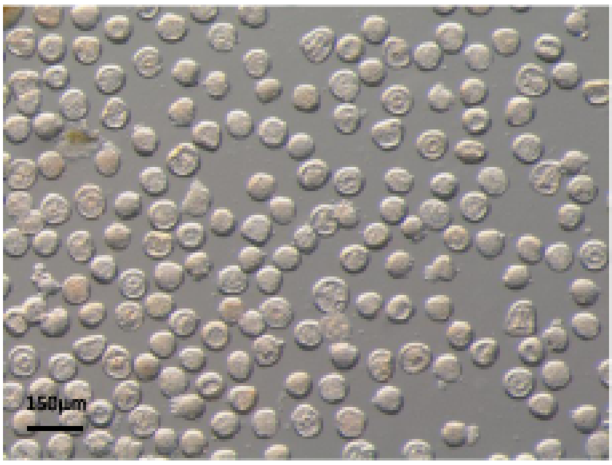

P.lividus embryos from parentals exposed to Cd H at 24hpf

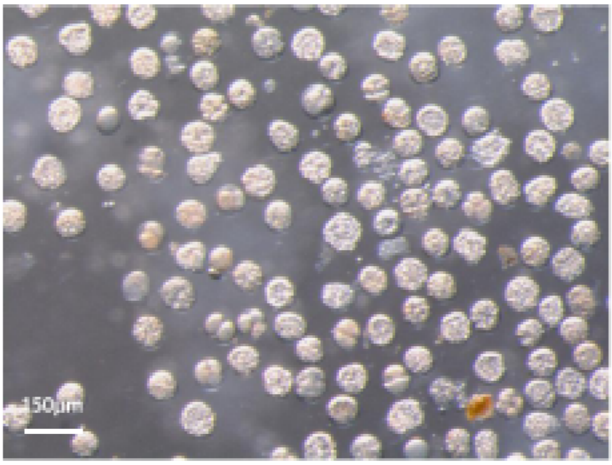

P.lividus embryos from parentals exposed to Zn L at 24 hpf

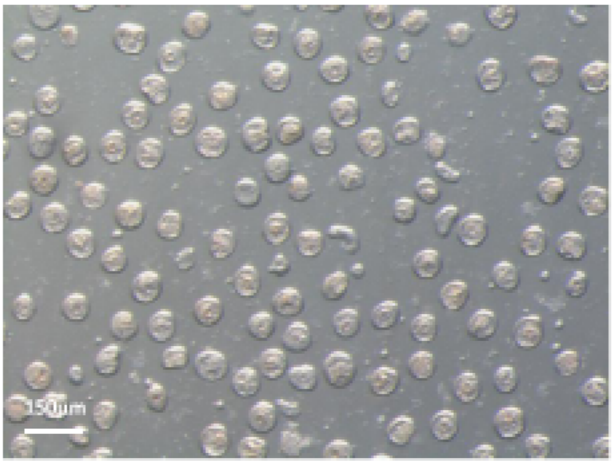

P.lividus embryos from parentals exposed to Zn L at 48 hpf

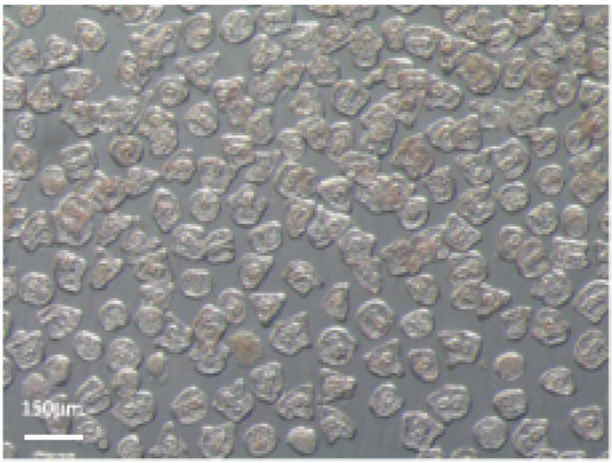

P.lividus embryos from parentals exposed to Zn H at 24hpf

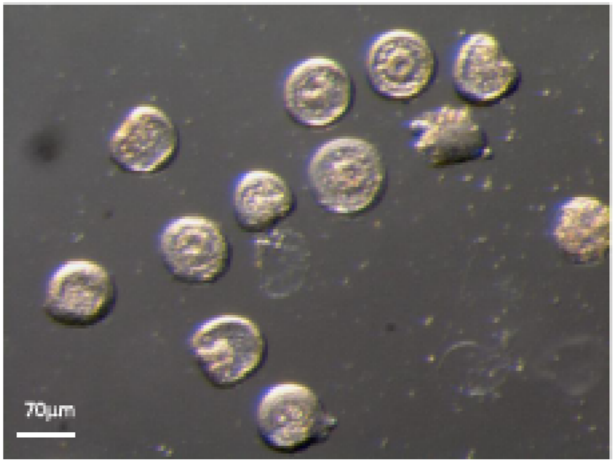

P.lividus embryos from parentals exposed to Zn H at 48hpf

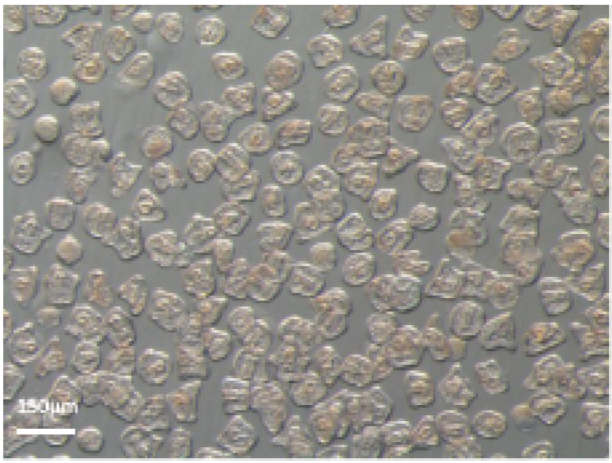

P.lividus embryos from parentals exposed to Mix L at 24 hpf

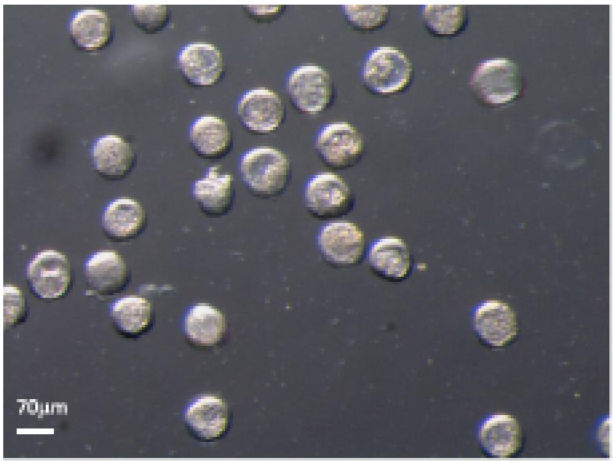

P.lividus embryos from parentals exposed to Mix L at 48 hpf

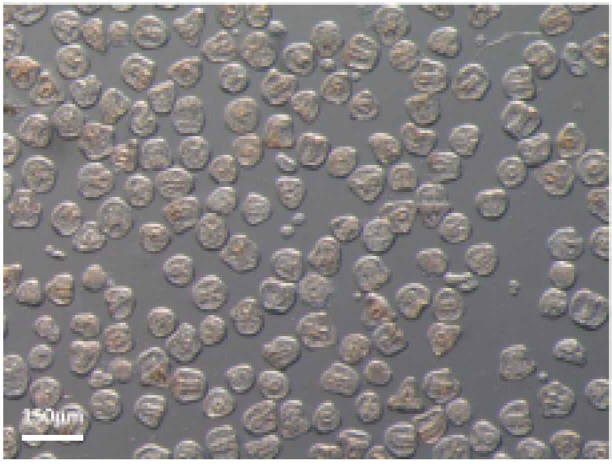

Supplement: Supplementary file 1 [file biology-10-00103-s001.pdf]
